# An Integrative Review of the Influence of Expectancies on Pain

**DOI:** 10.3389/fpsyg.2016.01270

**Published:** 2016-08-23

**Authors:** Kaya J. Peerdeman, Antoinette I. M. van Laarhoven, Madelon L. Peters, Andrea W. M. Evers

**Affiliations:** ^1^Health, Medical and Neuropsychology Unit, Leiden UniversityLeiden, Netherlands; ^2^Leiden Institute for Brain and Cognition, Leiden UniversityLeiden, Netherlands; ^3^Department of Psychiatry, Leiden University Medical CenterLeiden, Netherlands; ^4^Department of Clinical Psychological Science, Maastricht UniversityMaastricht, Netherlands

**Keywords:** pain, expectancy, self-efficacy, optimism, hope, trust, worry, pain catastrophizing

## Abstract

Expectancies can shape pain experiences. Attention for the influence of expectancies on pain has increased particularly due to research on placebo effects, of which expectancy is believed to be the core mechanism. In the current review, we provide a brief overview of the literature on the influence of expectancies on pain. We first discuss the central role of expectancy in the major psychological learning theories. Based on these theories, different kinds of expectancies can be distinguished. Pain experiences are influenced particularly by response expectancies directly pertaining to the pain experience itself, but can also be affected by self-efficacy expectancies regarding one’s ability to cope with pain, and possibly by stimulus expectancies regarding external events. These different kinds of expectancies might interact with each other, and related emotions and cognitions, as reflected by various multifaceted constructs in which expectancies are incorporated. Optimism and pain catastrophizing, in particular, but also hope, trust, worry, and neuroticism have been found to be associated with pain outcomes. We conclude with recommendations for further advancing research on the influence of expectancies on pain and for harnessing expectancy effects in clinical practice.

## Introduction

Pain is an unpleasant experience, in which not only sensory input but also psychological factors such as cognitions and emotions are at play. One important cognitive factor that can shape pain experiences is expectancies (i.e., cognitions regarding the probability of future experiences, events, and behavior; Mondloch et al., 2001; Rasmussen et al., 2009; Haanstra et al., 2012). The influence of expectancies on pain gained scientific interest especially due to research on placebo effects. A sham treatment such as a sugar pill or saline injection may relieve pain due to the mere expectation that a treatment will be helpful (i.e., placebo effect), or worsen pain when harmful treatment effects are expected (i.e., nocebo effect; Kirsch, 1985, 1997; Benedetti, 2014; Horing et al., 2014). Similarly, expectancies about treatment outcomes can enhance or reduce the analgesic effects of active treatments (e.g., Kam-Hansen et al., 2014; Aslaksen et al., 2015). Besides expectancies about the effects of treatment on pain, people can hold other kinds of expectancies. For example, someone might have high expectations about his/her ability to tolerate pain, and this might actually result in higher pain tolerance (Bandura, 1977; Litt, 1988). Different expectancies are likely to interact with each other, and with related emotions and cognitions. An understanding of the influence of expectancies on the experience of pain is crucial for both clinicians and researchers who treat or study pain, in order to obtain a comprehensive picture of the factors that determine pain and to optimize analgesic interventions via expectancy interventions.

In the current review, we provide a brief overview of the literature on the influence of expectancies on pain. First, we discuss the major psychological learning theories concerning expectancies. Based on these theories, different kinds of expectancies are distinguished, and we evaluate the influence of each of these on pain. Subsequently, we discuss multifaceted constructs (e.g., optimism, trust, and worry) in which expectancies are incorporated, and explore the evidence for their associations with pain. We conclude with recommendations for further research on the influence of expectancies on pain and for harnessing expectancy effects in clinical practice.

## Expectancies in Psychological Learning Theories

Expectancies are seen as important determinants of behavior, events, and experiences in many psychological theories of learning. Here we describe the most influential learning theories chronologically to gain an understanding of the conceptualization of expectancies.

One of the oldest and most systematically studied learning phenomena in psychology is conditioning. Classical conditioning is generally described as learning that results from pairing an initially neutral stimulus or event with a biologically relevant stimulus or event (Rescorla, 1988). In operant (or instrumental) conditioning, an association is made between a particular behavior and its consequence (e.g., reward or punishment; Bolles, 1972). According to most contemporary learning theorists, what is learned from these contingencies is outcome expectancies (although conditioning can also be automatic, i.e., not mediated cognitively; Pavlov, 1927; Bolles, 1972; Rescorla, 1988; Kirsch et al., 2004; Stewart-Williams and Podd, 2004). These expectancies indicate the perceived likelihood of a stimulus (e.g., receiving food) as the outcome of another stimulus or event (e.g., flashing of a light; in case of classical conditioning), or as the outcome of a specific behavior (e.g., pulling a lever; in case of operant conditioning; Pavlov, 1927; Bolles, 1972; Rescorla, 1988; Kirsch et al., 2004). These outcome expectancies are seen as important determinants of behavior. Since most of the expected outcomes described in conditioning research were external stimuli or events, these expectancies have been more specifically referred to as stimulus expectancies, to distinguish them from expectancies of other kinds of outcomes (specifically response expectancies regarding internal experiences, see below; Kirsch, 1985, 1997). In relation to pain, stimulus expectancies could for example entail expectations of the timing of a painful event, or of receiving a prescription for an analgesic on consulting a doctor.

Social learning theories were developed to address learning in interpersonal contexts and suggested that learning takes place not only via direct experiences (i.e., conditioning), but also via observation of others (i.e., observational learning), and verbal instructions (i.e., instructional learning; Bandura, 1977; Kirsch, 1985). Moreover, these theories postulate that not only outcome expectancies, but also other cognitions influence behavior. In the first major social learning theory, Rotter (1954) stated that the crucial determinant of behavior is the expected outcome of that behavior, in concert with the value a person places on that outcome. This theory had a major impact and has been further developed by many researchers. One of the most influential extensions is Bandura’s self-efficacy theory (Bandura, 1977). Bandura theorized that behavior is determined not only by expected outcomes, but also by expectancies regarding the ability to perform the behavior, i.e., self-efficacy expectancies. For example, someone with high self-efficacy expectations of tolerating pain might engage in physical activities despite pain (e.g., lifting heavy bags despite lower-back pain).

The theories described above focus mainly on expectancies of external outcomes and behavior (Rotter, 1954; Bolles, 1972; Bandura, 1977), expectancies of automatic, non-volitional responses – i.e., internal experiences such as emotions, and physical sensations such as pain – were largely overlooked. This was addressed by Kirsch (1985, 1997) in response expectancy theory. The hypothesis underlying response expectancy theory is that the expectation of one’s own automatic response to a certain behavior or situation (i.e., response expectancy, a form of outcome expectancy) not only influences behavior, but also directly influences one’s actual non-volitional response, and is as such directly self-confirming (Kirsch, 1985, 1997). These response expectancies are thought to be acquired through conditioning, instructional learning, and observational learning (Kirsch, 1985, 1997). An example of response expectancy is a patient’s expectation of pain relief upon taking an analgesic.

Based on these learning theories, in line with Kirsch’s conceptualization (Kirsch, 1985, 1997), we distinguish different kinds of expectancies: (1) outcome expectancies, which can be further subdivided into (a) stimulus expectancies, i.e., expectancies regarding external stimuli or events and (b) response expectancies, i.e., expectancies regarding internal non-volitional experiences; and (2) self-efficacy expectancies, i.e., expectancies regarding the ability to perform behavior. Several other, largely overlapping, typologies of expectancies have been proposed in the literature (e.g., Thompson and Sunol, 1995; Atlas and Wager, 2012), but since stimulus, response, and self-efficacy expectancies have the strongest theoretical foundation and empirical support, we focus only on these three kinds of expectancies in the current review.

## The Influence of Different Kinds of Expectancies on Pain

The different kinds of expectancies may influence pain in unique ways. Response expectancies probably exert the strongest and most direct influence on pain, since they can directly pertain to pain experiences. It is these kinds of expectancies that are generally believed to be the core mechanism of placebo and nocebo effects and that are consequently thought to greatly contribute to the efficacy of active treatments (Kirsch, 1997; Benedetti, 2014; Horing et al., 2014). When placebo or nocebo effects are induced, pain expectations are modified, and these response expectations predict changes in the intensity and unpleasantness of both experimental and clinical pain (Atlas et al., 2012; Schmid et al., 2013; Kirsch et al., 2014; Colagiuri et al., 2015; Peerdeman et al., 2016). Stimulus expectancies may exert an indirect influence on pain experiences, e.g., by affecting behavior, but could possibly also influence pain directly. Stimulus expectancies have received little scientific attention in the context of pain. There are indications that induced expectations regarding the timing of a painful event can reduce pain unpleasantness but not pain intensity (Price et al., 1980), but further research is needed. Self-efficacy expectancies have received much more scientific interest. They have consistently been found to predict pain coping efforts and pain tolerance (e.g., Litt, 1988; Jensen et al., 1991). Furthermore, self-efficacy expectancies have been found to be robust correlates of chronic pain severity (Jackson et al., 2014), and inducing self-efficacy can reduce experienced pain (e.g., Vancleef and Peters, 2011).

**FIGURE 1:**
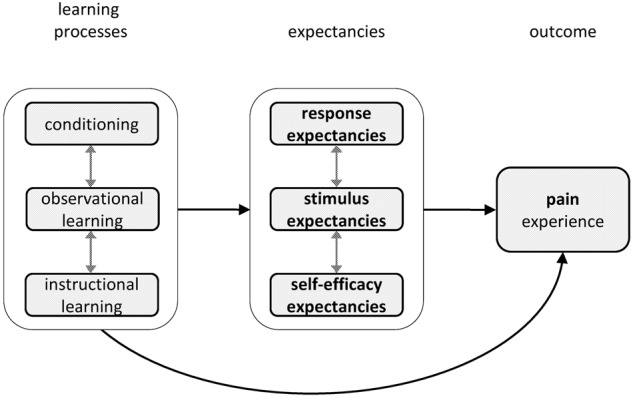
**Schematic depiction of the influence of expectancies on pain, including the learning processes that influence these expectancies.** Probable mediators and/or moderators are behavior, emotions, and cognitions. All elements in the model may also feed back to preceding elements.

Thus, empirical research supports the independent effects of response, stimulus, and self-efficacy expectancies on pain. These different kinds of expectancies may also interact with each other. For example, when inducing self-efficacy expectancies, response expectancies may also be enhanced (e.g., Vancleef and Peters, 2011), and effects of outcome expectancies may be mitigated if one has low self-efficacy expectancies, e.g., when one expects that a physical exercise will reduce neck pain, but also expects that one is not able to perform the exercise (e.g., Bandura, 1977). A schematic overview of the influence of the different kinds of expectancies on pain is depicted in **Figure 1.**

## Multifaceted Expectancy Constructs and Their Influence on Pain

The co-occurrence of different kinds of expectancies with related emotions and cognitions is captured in multifaceted constructs, in which expectancies are incorporated. Here we provide an overview of the most common multifaceted expectancy constructs and their associations with pain.

Optimism and hope are perhaps the most commonly considered multifaceted expectancy constructs. Optimism entails generalized positive expectancies of both stimulus and response type outcomes and is generally seen as a dispositional characteristic, although it can also vary depending on specific situations (Scheier and Carver, 1987). High levels of optimism are reliably associated with better health, including less severe acute and chronic pain (Rasmussen et al., 2009; Goodin and Bulls, 2013). The experimental induction of optimism can reduce pain sensitivity and pain interference (Hanssen et al., 2013; Boselie et al., 2014). Furthermore, optimism has been found to be associated with larger placebo analgesic effects (Geers et al., 2007, 2010; Morton et al., 2009; but see e.g., Hanssen et al., 2014). Hope is a related concept that is described as goal-directed thinking based on constructs that resemble outcome and self-efficacy expectancies (i.e., agency and pathway thinking, respectively) as well as motivational constructs (Snyder et al., 1991). Hope can pertain to specific situations or goals, but people also vary in their general tendency to be hopeful (Snyder, 2002). Several studies indicate that more hope is associated with using more pain-coping strategies, with higher pain tolerance, and with lower pain intensity (Snyder, 2002; Snyder et al., 2005; Rawdin et al., 2013). In addition, a hope-based intervention has been found to increase pain tolerance, though it did not affect pain intensity or pain threshold (Berg et al., 2008).

At an interpersonal level, trust is a multifaceted expectancy construct that is especially relevant in a medical context in which one has to entrust care of one’s health to another person (Hall et al., 2001). In the majority of definitions of trust, trusting is seen as entailing expectations that someone, e.g., the physician, will act in a benevolent manner, and that one can rely on this person and his/her intentions (Rotter, 1967; Pearson and Raeke, 2000; Hall et al., 2001). Trust takes on an emotional quality that extends beyond mere estimations of the likelihood of another person’s behaviors (Hall et al., 2001). Trust has been found to be associated with health behaviors such as adherence to treatment recommendations (Hall et al., 2001). In addition, trust in the physician has been associated with higher tolerance for treatment-induced pain (Caterinicchio, 1979).

Other constructs in which expectancies play a role and that can affect pain are constructs related to negative expectancies and the related emotions of fear and anxiety, such as worrying, pain catastrophizing, and neuroticism. Worrying is a repetitive thinking style that concerns a negative future (Borkovec et al., 1983). A person’s expectation that the event worried about will happen appears to be an important component of worrying (Butler and Mathews, 1983; Macleod et al., 1991). Furthermore, worrying has been suggested to heighten vigilance to threat, such as pain (Borkovec et al., 1983; Aldrich et al., 2000). Worrying about pain and worry intensity have been associated with higher pain levels and more frequent pain complaints, respectively (Verkuil et al., 2012; Davis et al., 2014). One interventional study, for example, found that a worry postponement intervention reduced somatic health complaints, including pain (Brosschot and van der Doef, 2006). The related construct of pain catastrophizing has frequently been a focus in pain research. Individuals who catastrophize often have negative response expectancies (e.g., that the pain may not go away), feel helpless about controlling their pain (i.e., low self-efficacy expectancies), are anxious, and worry and/or ruminate about their pain (Sullivan et al., 1995; Quartana et al., 2009). Pain catastrophizing is thus a comprehensive construct that involves different kinds of negative expectancies and related cognitions and emotions. Pain catastrophizing has consistently been linked to higher acute and chronic pain intensity, pain-related disability, and distress (e.g., Quartana et al., 2009; Wertli et al., 2014). The manipulation of pain catastrophizing has been found to affect experimental and chronic pain (both intensity and unpleasantness), though the findings are not fully consistent (Severeijns et al., 2005; Terry et al., 2015; Kjøgx et al., 2016). A last related construct is neuroticism. People high on neuroticism tend to be preoccupied with things that might go wrong (i.e., they tend to have negative expectancies, particularly negative outcome expectancies), to be easily frightened, and to feel despondent (Sanderman et al., 1995). Higher levels of neuroticism have been found to predict pain (Vassend et al., 2013; Wilner et al., 2014). Neuroticism has also been associated with placebo responses, but the results are equivocal (van Laarhoven et al., 2011; Darragh et al., 2014; Peerdeman et al., 2015).

## Implications of Current Findings

In the current review we set out to provide a brief overview of the literature on the influence of expectancies on pain. We found that different kinds of expectancies can be distinguished, which illustrates the complexity of the construct of expectancy. Nonetheless, it is clear that expectancies have an important influence on pain. Pain is influenced particularly by response expectancies that directly pertain to the pain experience itself. In addition, pain can be affected by self-efficacy expectancies regarding one’s ability to cope with pain and possibly also by stimulus expectancies regarding external events. The co-occurrence of various expectancies, and related emotions and cognitions, is captured by multifaceted constructs in which expectancies are incorporated. Optimism and pain catastrophizing, in particular, but also hope, trust, worry, and neuroticism have been found to be associated with pain.

To truly grasp the influence of expectancies on pain and to harness these effects, we recommend to refine existing theoretical models of expectancies by also addressing the interplay between different kinds of expectancies. Studies testing the predictions following from these models, should then assess multiple kinds of expectancies and expectancy constructs to determine their independent and interactive influence on pain. In this research the expectancy constructs of interest should be carefully determined, and clearly operationalized and reported. Since no single study can assess all kinds of expectancies, meta-analytic research can ultimately be used to make overarching inferences about the relative, and possible additive and interactive effects of the various kinds of expectancies on pain.

When addressing the effects of expectancies on pain in research and clinical practice, several additional considerations are of importance. First, it is important to take into account the strength and valence of the expectancy, as well as the intensity, nature, and duration of pain (Bandura, 1977; Kirsch, 1985, 1997; Peerdeman et al., 2016). For example, negative expectancies may exert larger effects on pain than positive expectancies (Baumeister et al., 2001), and acute pain is more sensitive to expectation interventions than chronic pain (Peerdeman et al., 2016). Second, research has generally focused on short-term effects in artificial laboratory situations. Although there are indications that expectancies can have an enduring clinical impact (e.g., Rodriguez-Raecke et al., 2010), further research into long-term effects is required. Third, expectancies are generally hypothesized and observed to have congruent effects on experiences: one experiences what one expects (Mondloch et al., 2001; Rasmussen et al., 2009; Haanstra et al., 2012; Peerdeman et al., 2016). However, in the case of a large discrepancy between what is expected and what is observed, expectancies may actually have detrimental effects, resulting in disappointment and experiences that contrast rather than mirror prior expectancies (Wilson et al., 1989; Thompson and Sunol, 1995; Geers and Lassiter, 1999; Shepperd et al., 2015). Importantly, if there is a large discrepancy between the expected and the actual outcome, the current experience may have a larger impact on learning (and thus on future expectancies and experiences), than if the actual experiences are in line with what was expected (Rescorla and Wagner, 1972). Thus, physicians should be wary of inducing either overly positive or overly negative expectancies regarding analgesic treatment outcomes in their patients.

Clinical applications of expectancy interventions are very promising for optimizing analgesic treatment effects. Several interventions tap into the learning processes that have been described in the learning theories (i.e., conditioning, observational, and instructional learning). Instructional learning via positive verbal suggestions of analgesic treatment outcomes, in particular, has been found to effectively reduce pain in clinical samples (Peerdeman et al., 2016). This demonstrates the significance of the information a physician provides when administering an analgesic treatment. A physician can address conditioning processes by assessing previous treatment experiences. If a treatment has previously been experienced as effective, current treatment outcomes could be enhanced by using the same route of treatment administration, while a switch (e.g., from topical to oral administration) may be beneficial if a patient’s previous experiences have been negative (Hofmann et al., 2014). Beneficial social learning may be facilitated via, for example, meetings with fellow or former patients or online video tutorials (Hunter et al., 2014). Furthermore, interventions evoking indirect experiences of pain reduction via mental imagery appear promising for inducing analgesia (Peerdeman et al., review). Experimental research suggests that the combination of multiple strategies, tapping into multiple learning processes (e.g., both conditioning and instructional learning), may be most beneficial (e.g., Amanzio and Benedetti, 1999; Peerdeman et al., review).

## Conclusion

The theoretical and empirical literature indicates that expectancies are an important determinant of pain, and that expectation interventions can effectively reduce pain. Future research requires the simultaneous study of different expectancy constructs in experimental and long-term interventional research, to further enhance our understanding of expectancies and their potential for optimizing analgesic interventions.

## Author Contributions

All authors contributed substantially to the conception and design of the work and to drafting and revising the work critically for important intellectual content. All authors have provided final approval of the submitted manuscript and agree to be accountable for all aspects of the work.

## Conflict of Interest Statement

The authors declare that the research was conducted in the absence of any commercial or financial relationships that could be construed as a potential conflict of interest.

## References

[B1] Aldrich S. Eccleston C. Crombez G. (2000). Worrying about chronic pain: vigilance to threat and misdirected problem solving. *Behav. Res. Ther.* 38 457–470. 10.1016/S0005-7967(99)00062-5 10816905

[B2] Amanzio M. Benedetti F. (1999). Neuropharmacological dissection of placebo analgesia: expectation-activated opioid systems versus conditioning-activated specific subsystems. *J. Neurosci.* 19 484–494. 9870976 10.1523/JNEUROSCI.19-01-00484.1999 PMC6782391

[B3] Aslaksen P. M. Zwarg M. L. Eilertsen H. I. H. Gorecka M. M. Bjorkedal E. (2015). Opposite effects of the same drug: reversal of topical analgesia by nocebo information. *Pain* 156 39–46. 10.1016/j.pain.0000000000000004 25599299

[B4] Atlas L. Y. Wager T. D. (2012). How expectations shape pain. *Neurosci. Lett.* 520 140–148. 10.1016/j.neulet.2012.03.039 22465136

[B5] Atlas L. Y. Whittington R. A. Lindquist M. A. Wielgosz J. Sonty N. Wager T. D. (2012). Dissociable influences of opiates and expectations on pain. *J. Neurosci.* 32 8053–8064. 10.1523/JNEUROSCI.0383-12.2012 22674280 PMC3387557

[B6] Bandura A. (1977). Self-efficacy: toward a unifying theory of behavioral change. *Psychol. Rev.* 84 191–215. 10.1037/0033-295X.84.2.191 847061

[B7] Baumeister R. F. Bratslavsky E. Finkenauer C. Vohs K. D. (2001). Bad is stronger than good. *Rev. Gen. Psychol.* 5 323–370. 10.1037/1089-2680.5.4.323

[B8] Benedetti F. (2014). *Placebo Effects.* Oxford: Oxford University Press.

[B9] Berg C. J. Snyder C. R. Hamilton N. (2008). The effectiveness of a hope intervention in coping with cold pressor pain. *J. Health Psychol.* 13 804–809. 10.1177/1359105308093864 18697893

[B10] Bolles R. C. (1972). Reinforcement, expectancy, and learning. *Psychol. Rev.* 79 394–409. 10.1037/h0033120

[B11] Borkovec T. D. Robinson E. Pruzinsky T. Depree J. A. (1983). Preliminary exploration of worry: some characteristics and processes. *Behav. Res. Ther.* 21 9–16. 10.1016/0005-7967(83)90121-3 6830571

[B12] Boselie J. J. L. M. Vancleef L. M. G. Smeets T. Peters M. L. (2014). Increasing optimism abolishes pain-induced impairments in executive task performance. *Pain* 155 334–340. 10.1016/j.pain.2013.10.014 24145210

[B13] Brosschot J. F. van der Doef M. (2006). Daily worrying and somatic health complaints: testing the effectiveness of a simple worry reduction intervention. *Psychol. Health* 21 19–31. 10.1080/14768320500105346

[B14] Butler G. Mathews A. (1983). Cognitive-processes in anxiety. *Adv. Behav. Res. Ther.* 5 51–62. 10.1016/0146-6402(83)90015-2

[B15] Caterinicchio R. P. (1979). Testing plausible path models of interpersonal-trust in patient-physician treatment relationships. *Soc. Sci. Med. Med. Psychol Med. Sociol.* 13A, 81–99. 10.1016/0271-7123(79)90011-7 551529

[B16] Colagiuri B. Quinn V. F. Colloca L. (2015). Nocebo hyperalgesia, partial reinforcement, and extinction. *J. Pain* 16 995–1004. 10.1016/j.jpain.2015.06.012 26168876

[B17] Darragh M. Booth R. J. Consedine N. S. (2014). Investigating the placebo personality outside the pain paradigm. *J. Psychosom. Res.* 76 414–421. 10.1016/j.jpsychores.2014.02.011 24745784

[B18] Davis C. E. Stockstill J. W. Stanley W. D. Wu Q. (2014). Pain-related worry in patients with chronic orofacial pain. *J. Am. Dent. Assoc.* 145 722–730. 10.14219/jada.2014.37 24982278

[B19] Geers A. L. Kosbab K. Helfer S. G. Weiland P. E. Wellman J. A. (2007). Further evidence for individual differences in placebo responding: an interactionist perspective. *J. Psychosom. Res.* 62 563–570. 10.1016/j.jpsychores.2006.12.005 17467411

[B20] Geers A. L. Lassiter G. D. (1999). Affective expectations and information gain: evidence for assimilation and contrast effects in affective experience. *J. Exp. Soc. Psychol.* 35 394–413. 10.1006/jesp.1999.1377

[B21] Geers A. L. Wellman J. A. Fowler S. L. Helfer S. G. France C. R. (2010). Dispositional optimism predicts placebo analgesia. *J. Pain* 11 1165–1171. 10.1016/j.jpain.2010.02.014 20627818 PMC2956003

[B22] Goodin B. R. Bulls H. W. (2013). Optimism and the experience of pain: benefits of seeing the glass as half full. *Curr. Pain Headache Rep.* 17 329 10.1007/s11916-013-0329-8 PMC3935764 23519832

[B23] Haanstra T. M. Van Den Berg T. Ostelo R. W. Poolman R. W. Jansma E. P. Cuijpers P. (2012). Systematic review: do patient expectations influence treatment outcomes in total knee and total hip arthroplasty? *Health Qual. Life Outcomes* 10:152 10.1186/1477-7525-10-152 PMC3568025 23245187

[B24] Hall M. A. Dugan E. Zheng B. Y. Mishra A. K. (2001). Trust in physicians and medical institutions: what is it, can it be measured, and does it matter? *Milbank Q.* 79 613–639. 10.1111/1468-0009.00223 11789119 PMC2751209

[B25] Hanssen M. M. Peters M. L. Vlaeyen J. W. S. Meevissen Y. M. C. Vancleef L. M. G. (2013). Optimism lowers pain: evidence of the causal status and underlying mechanisms. *Pain* 154 53–58. 10.1016/j.pain.2012.08.006 23084002

[B26] Hanssen M. M. Vancleef L. M. G. Vlaeyen J. W. S. Peters M. L. (2014). More optimism, less pain! The influence of generalized and pain-specific expectations on experienced cold-pressor pain. *J. Behav. Med.* 37 47–58. 10.1007/s10865-012-9463-8 23239369

[B27] Hofmann M. Wrobel N. Kessner S. Bingel U. (2014). Minimizing carry-over effects after treatment failure and maximizing therapeutic outcome: can changing the route of administration mitigate the influence of treatment history? *Z. Psychol.* 222 171–178. 10.1027/2151-2604/a000180

[B28] Horing B. Weimer K. Muth E. R. Enck P. (2014). Prediction of placebo responses: a systematic review of the literature. *Front. Psychol.* 5:1079 10.3389/fpsyg.2014.01079 PMC4181242 25324797

[B29] Hunter T. Siess F. Colloca L. (2014). Socially induced placebo analgesia: a comparison of a pre-recorded versus live face-to-face observation. *Eur. J. Pain* 18 914–922. 10.1002/j.1532-2149.2013.00436.x 24347563 PMC4061280

[B30] Jackson T. Wang Y. L. Wang Y. Fan H. Y. (2014). Self-efficacy and chronic pain outcomes: a meta-analytic review. *J. Pain* 15 800–814. 10.1016/j.jpain.2014.05.002 24878675

[B31] Jensen M. P. Turner J. A. Romano J. M. (1991). Self-efficacy and outcome expectancies: relationship to chronic pain coping strategies and adjustment. *Pain* 44 263–269. 10.1016/0304-3959(91)90095-F 2052395

[B32] Kam-Hansen S. Jakubowski M. Kelley J. M. Kirsch I. Hoaglin D. C. Kaptchuk T. J. (2014). Altered placebo and drug labeling changes the outcome of episodic migraine attacks. *Sci. Transl. Med.* 6:218ra215 10.1126/scitranslmed.3006175 PMC4005597 24401940

[B33] Kirsch I. (1985). Response expectancy as a determinant of experience and behavior. *Am. Psychol.* 40 1189–1202. 10.1037/0003-066X.40.11.1189

[B34] Kirsch I. (1997). Response expectancy theory and application: a decennial review. *Appl. Prev. Psychol.* 6 69–79. 10.1016/S0962-1849(05)80012-5

[B35] Kirsch I. Kong J. Sadler P. Spaeth R. Cook A. Kaptchuk T. (2014). Expectancy and conditioning in placebo analgesia: separate or connected processes? *Psychol. Conscious.* 1 51–59. 10.1037/cns0000007 PMC4118664 25093194

[B36] Kirsch I. Lynn S. J. Vigorito M. Miller R. R. (2004). The role of cognition in classical and operant conditioning. *J. Clin. Psychol.* 60 369–392. 10.1002/jclp.10251 15022268

[B37] Kjøgx H. Kasch H. Zachariae R. Svensson P. Jensen T. S. Vase L. (2016). Experimental manipulations of pain catastrophizing influence pain levels in patients with chronic pain and healthy volunteers. *Pain* 157 1287–1296. 10.1097/j.pain.0000000000000519 26871534

[B38] Litt M. D. (1988). Self-efficacy and perceived control: cognitive mediators of pain tolerance. *J. Pers. Soc. Psychol.* 54 149–160. 10.1037/0022-3514.54.1.149 3346804

[B39] Macleod A. K. Williams J. M. G. Bekerian D. A. (1991). Worry is reasonable: the role of explanations in pessimism about future personal events. *J. Abnorm. Psychol.* 100 478–486. 10.1037/0021-843X.100.4.478 1757661

[B40] Mondloch M. V. Cole D. C. Frank J. W. (2001). Does how you do depend on how you think you’ll do? A systematic review of the evidence for a relation between patients’ recovery expectations and health outcomes. *Can. Med. Assoc. J.* 165 174–179. 11501456 PMC81284

[B41] Morton D. L. Watson A. El-Deredy W. Jones A. K. P. (2009). Reproducibility of placebo analgesia: effect of dispositional optimism. *Pain* 146 194–198. 10.1016/j.pain.2009.07.026 19692178

[B42] Pavlov I. P. (1927). *Conditioned Reflexes: An Investigation of the Physiological Activity of the Cerebral Cortex.* London: Oxford University Press. 10.5214/ans.0972-7531.1017309 PMC4116985 25205891

[B43] Pearson S. D. Raeke L. H. (2000). Patients’ trust in physicians: many theories, few measures, and little data. *J. Gen. Intern. Med.* 15 509–513. 10.1046/j.1525-1497.2000.11002.x 10940139 PMC1495476

[B44] Peerdeman K. J. Van Laarhoven A. I. M. Donders A. R. T. Hopman M. T. E. Peters M. L. Evers A. W. M. (2015). Inducing expectations for health: effects of verbal suggestion and imagery on pain, itch, and fatigue as indicators of physical sensitivity. *PLoS ONE* 10:e0139563 10.1371/journal.pone.0139563 PMC4598027 26448183

[B45] Peerdeman K. J. Van Laarhoven A. I. M. Keij S. M. Vase L. Rovers M. M. Peters M. L. (2016). Relieving patients’ pain with expectation interventions: a meta-analysis. *Pain* 157 1179–1191. 10.1097/j.pain.0000000000000540 26945235

[B46] Price D. D. Barrell J. J. Gracely R. H. (1980). A psychophysical analysis of experiential factors that selectively influence the affective dimension of pain. *Pain* 8 137–149. 10.1016/0304-3959(88)90001-2 7402678

[B47] Quartana P. J. Campbell C. M. Edwards R. R. (2009). Pain catastrophizing: a critical review. *Expert Rev. Neurother.* 9 745–758. 10.1586/ern.09.34 19402782 PMC2696024

[B48] Rasmussen H. N. Scheier M. F. Greenhouse J. B. (2009). Optimism and physical health: a meta-analytic review. *Ann. Behav. Med.* 37 239–256. 10.1007/s12160-009-9111-x 19711142 PMC2941870

[B49] Rawdin B. Evans C. Rabow M. W. (2013). The relationships among hope, pain, psychological distress, and spiritual well-being in oncology outpatients. *J. Palliat. Med.* 16 167–172. 10.1089/jpm.2012.0223 23101471 PMC3569921

[B50] Rescorla R. A. (1988). Pavlovian conditioning. It’s not what you think it is. *Am. Psychol.* 43 151–160. 3364852 10.1037//0003-066x.43.3.151

[B51] Rescorla R. A. Wagner A. R. (1972). “A theory of Pavlovian conditioning: variations in the effectiveness of reinforcement and nonreinforcement,” in *Classical Conditioning II: Current Research and Theory* eds Black A. H. Prokasy W. F. (New York, NY: Appleton-Century-Crofts) 64–99.

[B52] Rodriguez-Raecke R. Doganci B. Breimhorst M. Stankewitz A. Buchel C. Birklein F. (2010). Insular cortex activity is associated with effects of negative expectation on nociceptive long-term habituation. *J. Neurosci.* 30 11363–11368. 10.1523/JNEUROSCI.2197-10.2010 20739557 PMC6633339

[B53] Rotter J. B. (1954). *Social Learning and Clinical Psychology.* New York, NY: Prentice-Hall.

[B54] Rotter J. B. (1967). A new scale for the measurement of interpersonal trust. *J. Pers.* 35 651–665. 10.1111/j.1467-6494.1967.tb01454.x 4865583

[B55] Sanderman R. Arrindell W. A. Ranchor A. V. Eysenck H. J. Eysenck S. B. G. (1995). *Het Meten van Persoonlijkheidskenmerken met de Eysenck Personality Questionnaire (EPQ): Een Handleiding.* Groningen: Noordelijk Centrum voor Gezondheidsvraagstukken.

[B56] Scheier M. F. Carver C. S. (1987). Dispositional optimism and physical well-being: the influence of generalized outcome expectancies on health. *J. Pers.* 55 169–210. 10.1111/j.1467-6494.1987.tb00434.x 3497256

[B57] Schmid J. Theysohn N. Gass F. Benson S. Gramsch C. Forsting M. (2013). Neural mechanisms mediating positive and negative treatment expectations in visceral pain: a functional magnetic resonance imaging study on placebo and nocebo effects in healthy volunteers. *Pain* 154 2372–2380. 10.1016/j.pain.2013.07.013 23867733

[B58] Severeijns R. Van Den Hout M. A. Vlaeyen J. W. S. (2005). The causal status of pain catastrophizing: an experimental test with healthy participants. *Eur. J. Pain* 9 257–265. 10.1016/j.ejpain.2004.07.005 15862475

[B59] Shepperd J. A. Waters E. A. Weinstein N. D. Klein W. M. P. (2015). A primer on unrealistic optimism. *Curr. Dir. Psychol. Sci.* 24 232–237. 10.1177/0963721414568341 26089606 PMC4467896

[B60] Snyder C. R. (2002). Hope theory: rainbows in the mind. *Psychol. Inquiry* 13 249–275. 10.1207/S15327965PLI1304_01

[B61] Snyder C. R. Berg C. Woodward J. T. Gum A. Rand K. L. Wrobleski K. K. (2005). Hope against the cold: individual differences in trait hope and acute pain tolerance on the cold pressor task. *J. Pers.* 73 287–312. 10.1111/j.1467-6494.2005.00318.x 15745432

[B62] Snyder C. R. Harris C. Anderson J. R. Holleran S. A. Irving L. M. Sigmon S. T. (1991). The will and the ways: development and validation of an individual-differences measure of hope. *J. Pers. Soc. Psychol.* 60 570–585. 10.1037/0022-3514.60.4.570 2037968

[B63] Stewart-Williams S. Podd J. (2004). The placebo effect: dissolving the expectancy versus conditioning debate. *Psychol. Bull.* 130 324–340. 10.1037/0033-2909.130.2.324 14979775

[B64] Sullivan M. J. L. Bishop S. R. Pivik J. (1995). The pain catastrophizing scale: development and validation. *Psychol. Assess.* 7 524–532. 10.1037/1040-3590.7.4.524

[B65] Terry E. L. Thompson K. A. Rhudy J. L. (2015). Experimental reduction of pain catastrophizing modulates pain report but not spinal nociception as verified by mediation analyses. *Pain* 156 1477–1488. 10.1097/j.pain.0000000000000192 25887463

[B66] Thompson A. G. H. Sunol R. (1995). Expectations as determinants of patient satisfaction: concepts, theory and evidence. *Int. J. Qual. Health Care* 7 127–141. 10.1016/1353-4505(95)00008-J 7655809

[B67] van Laarhoven A. I. M. Vogelaar M. L. Wilder-Smith O. H. Van Riel P. L. C. M. Van De Kerkhof P. C. M. Kraaimaat F. W. (2011). Induction of nocebo and placebo effects on itch and pain by verbal suggestions. *Pain* 152 1486–1494. 10.1016/j.pain.2011.01.043 21353388

[B68] Vancleef L. M. G. Peters M. L. (2011). The influence of perceived control and self-efficacy on the sensory evaluation of experimentally induced pain. *J. Behav. Ther. Exp. Psychiatry* 42 511–517. 10.1016/j.jbtep.2011.05.006 21699876

[B69] Vassend O. Roysamb E. Nielsen C. S. (2013). Five-factor personality traits and pain sensitivity: a twin study. *Pain* 154 722–728. 10.1016/j.pain.2013.01.010 23473786

[B70] Verkuil B. Brosschot J. F. Meerman E. E. Thayer J. F. (2012). Effects of momentary assessed stressful events and worry episodes on somatic health complaints. *Psychol. Health* 27 141–158. 10.1080/08870441003653470 21038174

[B71] Wertli M. M. Burgstaller J. M. Weiser S. Steurer J. Kofmehl R. Held U. (2014). Influence of catastrophizing on treatment outcome in patients with nonspecific low back pain. *Spine (Phila Pa 1976)* 39 263–273. 10.1097/BRS.0000000000000110 24253796

[B72] Wilner J. G. Vranceanu A. M. Blashill A. J. (2014). Neuroticism prospectively predicts pain among adolescents: results from a nationally representative sample. *J. Psychosom. Res.* 77 474–476. 10.1016/j.jpsychores.2014.10.012 25466384 PMC4852549

[B73] Wilson T. D. Lisle D. J. Kraft D. Wetzel C. G. (1989). Preferences as expectation-driven inferences: effects of affective expectations on affective experience. *J. Pers. Soc. Psychol.* 56 519–530. 10.1037/0022-3514.56.4.519 2709307

